# Mental health issues associated with the management of tuberculosis in Homabay, Busia and Kakamega Counties, Kenya

**DOI:** 10.1371/journal.pone.0298268

**Published:** 2024-04-16

**Authors:** John Otieno, Albino Luciani, Sheila Lumumba, George Gikunda, Colleta Kiilu, Norah Ogutu, Bryson Sifuma, Dennis Kinyua, Diana Mukami, Catherine Mwenda, Aiban Ronoh, Yvonne Opanga

**Affiliations:** 1 Amref Health Africa Institute of Capacity Development, Nairobi, Kenya; 2 Kenya Medical Research Institute, Center for Global Health Research, Nairobi, Kenya; 3 Ministry of Health, National TB, Leprosy and Lung Diseases Program, Nairobi, Kenya; 4 Amref Health Africa in Kenya, Nairobi, Kenya; Kilimanjaro Clinical Research Institute, UNITED REPUBLIC OF TANZANIA

## Abstract

**Background:**

Despite the implementation of patient-centred care, mental health issues remain a significant risk factor and comorbidity for Tuberculosis (TB) disease. Mental health issues being co-morbidities to TB are likely to increase the disease burden of the affected population. This study therefore investigated the prevalence and impact of mental health issues in Tuberculosis (TB) patients.

**Methods:**

This cross-sectional study design used mixed methods in data collection. The study used structured questionnaires, key informant interviews and focus group discussions. 127 TB patients were purposively selected from a group of patients who previously recovered successfully, with a history of relapse or are currently on TB treatment in high-volume facilities in Homa Bay, Busia and Kakamega Counties. 30 Key informant interviews were conducted with Healthcare workers. Quantitative data analysis was done using STATA version 14. Thematic analysis was employed to analyze qualitative data using NVivo version 10.

**Results:**

The findings showed that the most common mental health issues affecting TB patients were anxiety (66%) and depression (55%), which were commonly experienced during the presumptive stage of TB. Qualitative data revealed that stigma was the main barrier that hindered TB patients from accessing care. TB patients articulated the benefits of support structures ranging from positive encouragement, reminders on taking drugs, accompaniment to the clinic, and financial support in TB management. Furthermore, the study established that the majority of Health Care Workers (HCWs) were not prepared to handle TB patients’ mental issues, a gap that is likely to impact the quality of care TB patients receive.

**Conclusion:**

The study established that mental health issues impact TB treatment outcomes. Healthcare systems should prioritize the integration of mental health care into TB programs to address the high prevalence of mental health issues among TB patients.

## 1 Introduction

Tuberculosis (TB) remains a major global public health issue and is one of the leading causes of death in Low and Middle-income countries [1]. In Kenya, the burden of TB is 558 per 100,000 population as of 2016 [2]. Kakamega, Busia and Homa Bay counties are located in the Western region of Kenya. These counties are marked with heterogeneous TB and Human Immunodeficiency Virus (HIV) epidemics, ranging from Homa Bay County (HIV prevalence, 20.7%; TB case notification rate 176–250 per 100,000 population), Busia (HIV Prevalence,7.7%; TB case notification rate, 60–100 per 100,000 population) and Kakamega (HIV prevalence,4.5%; TB case notification rate, 101–175 per 100,000 population) [3, 2]. The interaction between TB and HIV is bidirectional, with each disease exacerbating the progress of the other, leading to a higher burden of TB-HIV coinfection. People living with HIV are 16 times more likely to contract TB than people without HIV [4] and developing TB can accelerate the progression of HIV to Acquired Immunodeficiency Syndrome (AIDs) [5]. In 2021, about 187,000 people died of HIV-associated TB [4].

The burden of TB occurs in concert with different dimensions of mental health issues. Mental health issues are an emerging public health concern and have been reported to be a significant barrier to adherence to TB and multidrug-resistant (MDR) TB treatment [6]. While data on the prevalence of Mental health issues in Kenya is inadequate, a report by the Kenya National Commission on Human Rights (KNHCR) in 2011 estimated that up to 25% of outpatients and 40% of inpatients in health facilities will suffer from a mental health issue [7]. Depression and anxiety disorders are two of the most frequent diagnoses of mental health issues made in general hospital settings [8]. Depression is a mental disorder characterized by persistent sadness and a lack of interest or pleasure in previously rewarding or enjoyable activities [9]. Anxiety disorders are characterised by excessive fear and worry with symptoms that are severe enough to result in significant distress or significant impairment in functioning [10]. In 2019, 280 million people were living with depression and 301 million people were living with an anxiety disorder [10].

The prevalence of depression and anxiety as one of the most common mental health issues is very high among people with chronic diseases. Several studies have reported comorbidities with TB leading to poor quality of life which increases the mortality rate attributed to TB [11, 12]. Individuals with TB have elevated rates of depression and anxiety, which is one of the major causes of lower treatment success rate [13]. In addition, there is growing evidence that many medications used to treat tuberculosis can have significant adverse psychiatric effects and some medications, such as rifampicin, may reduce the effectiveness of antipsychotic drugs [13].

With the increase in the prevalence of mental health among TB patients, there is a risk of delays in care for TB as well as increased defaulter rates among patients on anti-TB medications. Over the past 5 years, access to effective mental health care has become more urgent as rates of non-adherence, Drug-resistant TB (DR-TB) and Multidrug and rifampicin-resistant (MDR/RR) TB have increased. According to Sweetland et al. [14], defaulting to taking the anti-TB medication increases the risk of treatment failure and drug resistance, increasing TB-related morbidity and mortality [14]. This risk creates a need for mental health interventions that can be delivered through primary care to be tested in the context of TB prevention and management to improve the clinical outcomes of the patients.

Informed by the growing evidence from programme reviews, studies, and surveys, including the startling findings of the 2016 TB prevalence survey, The Kenya National TB, Leprosy and Lung Disease Programme (KNTLD-P) has renewed its focus on strategies to find and successfully treat all people with TB disease. The National Strategic Plan (NSP) for Tuberculosis, Leprosy, and Lung Health 2019–2023 consolidates the learning from the last few years. It articulates a patient-centered approach to planning and evidence-based prioritization of resource allocation to close the gaps and accelerate progress to reaching and curing all people with TB. It envisages a multi-sectoral partnership where stakeholders bring their comparative advantage to bear in addressing systemic and root causes of the gaps along the TB patient pathway. The National Strategic Plan (NSP) recommendations strengthen the capacity of the Tuberculosis, Leprosy, and Lung Disease Programme in prevention, detection and response to Mental health while investing in TB patient support structures ranging from close follow-up, Counselling and peer support as well as training Health Care Workers to provide need-based care for TB patients in adherence with clinical guidelines for TB and mental health care.

In this study, we assessed how mental health issues experienced by TB patients affect TB treatment access and management in Homa Bay, Busia and Kakamega Counties. We explore the perspective of current and past TB patients and Health Care workers’ perception of their competencies and attitude in the context of Mental Healthcare.

## 2 Methodology

The study adopted a cross-sectional study design using mixed methods of data collection. Quantitative data was collected through TB patient surveys while qualitative data was collected using Focus Group Discussions (FGDs) and In-depth interviews for the Key Informant interviews. The study was conducted in Kakamega, Homabay and Busia counties, Kenya: The study targeted 73 TB patients (Kakamega n = 32, Homabay n = 22, Busia n = 19) for the patient survey.

The respondents were recruited from 6^th^ April 2022 to 10^th^ April 2022. The clients reporting to the TB clinic were systematically sampled (every 3rd client) to respond. The sample for the TB patient survey was drawn from 10% of all TB patients recruited in the selected health facilities in the 4 months preceding the survey as per the TIBU database. The sampling approach also included the length of treatment, the treatment outcome and demographic factors like age, sex, and level of education, among others that can be associated with more information and knowledgeability.

Purposive sampling was used to recruit respondents for FGDs. The respondents were sampled from the KNTLD-P DR-TB facility register. For current TB patients, the register was used to determine how many patients were scheduled for review. They were randomly selected, informed about the study and requested to join the FGDs. For respondents of the successfully recovered and relapsed FGDs, the register was used to obtain their telephone contacts. The identified respondents were contacted and requested to come to the facility on a certain day at a certain time for the FGDs. FGDs were conducted with 24 current TB patients, 12 successfully recovered patients and 18 relapsed TB patients. For the KIIs, respondents were purposively sampled from individuals who had direct knowledge of the key interventions. Key Informant Interviews were conducted with County health management teams, Sub-county health management teams and Healthcare workers in selected facilities. A total of 9 FGDs and 31 Key Informant Interviews (KIIs) were conducted.

The Inclusion Criteria for this study were current TB patients, successfully recovered TB patients, relapsed TB patients and TB management healthcare providers.

Table 1 shows the list of respondents that were interviewed:

**Table 1:** Sampling frame.

Level	Respondent	Number
Clients (questionnaire)	TB patients reporting to TB clinic	73
Clients (FGD)	Current TB patients	24
Clients (FGD)	Past TB patients-successful	12
Clients (FGD)	Current TB patients-relapsed	18
County (KII)	County TB Coordinator	3
	County Director of Health	1
	County Disease Surveillance Coordinator	1
	County Community Health Strategy focal person	2
	County Reproductive Health Coordinator	1
Sub-county (KII)	Sub-County TB Coordinator	5
	Sub-County Director of Health	2
	Sub-County Disease Surveillance Coordinator	2
	Sub-County Health Records & Information Officer	6
Amref	Amref Health Africa Staff	1
Facility	Health Facility In-Charges	3
	Healthcare workers	2
	Community Health Extension Worker (CHEW)	2

### Data management procedures

Quantitative data was cleaned, and a master data sheet was created. Analysis was done using STATA version 14. Descriptive statistics were used to summarize and identify patterns in the data and develop key statistics and tabulations that indicate differences in different subgroups.

Inferential statistics were used to explore the data further, with t-tests being used to compare the differences in means of different related groups of respondents.

The KII and FGD audios were transcribed, and the transcripts were generated and cleaned. The analysis of qualitative data was conducted using NVivo version 10 to extract codes. Subsequently, a thematic process was employed to identify subthemes and overarching themes. The resulting output was utilized in the report writing process and presented in narrative form.

All data collected during the study is the intellectual property of Amref Health Africa. Therefore, data will be stored and archived as per Amref data management policy.

### Ethical considerations and procedures undertaken

Ethical clearance was obtained from the Amref Ethics and Scientific Review Commission (ESRC P1138/2022). Further, a research permit was obtained from the National Commission for Science, Technology and Innovation (NACOSTI/P/22/16465). Permission was secured from the respective County and Sub-County health management teams. Verbal Informed consent was obtained from each study participant after providing information on the study’s objectives and the benefits/risks associated with their participation. Privacy and confidentiality were achieved by conducting the interviews in spaces that ensured privacy. The data collected was anonymized. Personal identification information was not reported.

## 3 Results

### Demographic characteristics of the respondents

The study interviewed 73 TB patients as shown in Table 2 of which 71% were male and 29% were female. More than half of the patients had primary-level education (58%) and were married (56%). The average age of the patients was 37.9%.

**Table 2:** Demographic profile of the patients.

	Characteristic	Overall (n = 73)
Sex of respondent	Male	71%
	Female	29%
Level of education	Never gone to school	4%
	Primary	58%
	Secondary	26%
	College	5%
	University	7%
Marital status	Married	56%
	Single	33%
	Divorced/ separated	8%
	Widowed/ Widower	3%
Age	Average	37.9

### TB diagnosis and treatment

The average length of time TB patients spent on TB treatment was 5.77 months as shown on S1 Fig. 11% of the TB patients interviewed reported that another household member had also been diagnosed with TB. 27% of the respondents had a case of recurrence of TB after treatment completion.

### Impact of TB Diagnosis on utilization of services

S2 Fig shows some of the negative impacts patients experienced after being diagnosed with TB. 41% mentioned that they experienced self-stigmatization after the TB diagnosis. 34% indicated non-adherence to anti-TB medication, and 32% cited delay in seeking treatment. Isolation, suicidal thoughts and self-harm and poor nutrition were cited by 29%, 19% and 18% of the patients, respectively.

The survey further assessed how stigma was a barrier to access and utilization of TB services. From S3 Fig, the most common effects of stigma included TB patients defaulting on treatment (62%), delay in seeking treatment (60%) and or relapse after being started on TB treatment (32%).

### Mental health issues affecting TB patients

The study sought to establish the mental health issues TB patients experience. Based on findings in S4 Fig, the two most common mental health issues affecting TB patients were anxiety, which was mentioned by two-thirds of TB patients (66%) and depression, as mentioned by 55% of respondents interviewed. Trauma-related disorder (16%), alcoholism (5%), psychosis (3%), drug abuse (1%) and tobacco use (1%) were the other mental health issues cited by TB patients. 19% of the TB patients reported that they experience no mental health issue.

S5 Fig shows the stages at which TB patients mostly experienced mental health issues. Mental health issues were most prevalent during the testing stage (40%) and at the stage when they suspected they may have TB (33%). Notably, at recovery, no respondent mentioned having experienced mental health issues.

Table 3 below shows an assessment of the severity of the mental health issue at each stage of the TB management pathway. Anxiety (56%) and depression (30%) were most common at the TB suspicion stage. Notably, there was a steady rise in the proportion of respondents who reported not experiencing any mental health issues, from 26% at the suspicion stage to 70% at recovery.

**Table 3:** Mental health issues experienced during the TB management pathway.

	Suspicion	Testing	Management	Recovery
**Anxiety**	56%	45%	21%	14%
**Depression**	30%	38%	22%	8%
**Psychosis**	3%	1%	1%	1%
**Drug abuse**	0%	0%	0%	0%
**Trauma-related disorder**	4%	4%	3%	0%
**Alcoholism**	5%	3%	1%	1%
**Tobacco-related**	0%	0%	0%	0%
**None**	26%	23%	53%	70%
**Don’t Know**	1%	1%	3%	11%

### Support received by TB patients when experiencing mental health issues

From S6 Fig, the most common support TB patients received from family/friends was encouragement (85%) followed by reminders about taking drugs (73%). Other ways of support mentioned by TB patients were being accompanied to the clinic (44%) and financial support (41%).

### Health care system and mental health of TB patients’ management

The survey sought to establish the existing support structures for TB patients with mental health issues. Findings in S7 Fig show that most TB patients received support from treatment centres (53%) and their families (52%). Support from CHVs came third and was mentioned by 33% of total respondents while 21% mentioned counselling centres and 15% stated support groups.

The study assessed ways in which community structures could be improved to enhance health-seeking behaviour among TB patients with mental health issues. The findings are displayed in S8 Fig. 78% stated the need to sensitize community members on mental health, 52% mentioned the establishment of counselling and treatment centres and 33% stated the need to integrate mental health into the TB case management process.

### Attitudes and perceptions of HCWs and contribution to mental health issues

The study assessed the effect of healthcare workers’ attitudes on TB patients. S9 Fig shows that the most common effects of a negative attitude from HCWs were poor quality of care (34%), loss of follow-up (27%) and stigmatization (18%). 38% of the TB patients were unaware of any effect due to HCWs’ attitudes.

Qualitative data was collected to further validate the quantitative findings. The qualitative analysis of the study revealed a nuanced perspective among respondents providing insight into the impact of mental health issues on the management of TB. Through in-depth interviews and thematic analysis, the following key themes and subthemes emerged.

### Theme 1: Psychological barriers to utilization of services

#### Subtheme 1: Stigma as a barrier to screening

FGD and KII respondents stated that stigmatization led to patients not disclosing their TB status to their families, increasing the risk of transmission among the household members and impacting morbidity rates of TB in the community.

“*Stigma prevents disclosure to family members since there is shame associated with having TB*.”*- Current patient, Busia*

“*TB is associated with witchcraft*; *this affects them in terms of seeking treatment services and adherence to treatment*. *It also affects their self-worth and interaction with others in the society”- Sub-county MOH Official-Ndhiwa*

#### Subtheme 2: Stigma as a barrier to utilization of services

The societal stigma coupled with the misconception concerning TB potentially leads to delay and non-adherence to treatment.

“*Stigma delays presenting yourself for screening when you suspect TB*.*”- Current patient*, *Busia*

“*Uneducated community members who mostly misinterpret and speculate negatively about TB spread stigma among the patients*, *this can lead to suicidal tendencies*, *depression*, *poor nutrition and high TB mortality rates in such communities”- Current patient*, *Homabay*

### Theme 2: Psychological impact and coping mechanisms

#### Subtheme 1: Depression and anxiety

KII respondents reported that the mental health issues faced by TB patients were depression, anxiety and stress. Depression was majorly associated with the length of treatment and was highlighted as a major concern among drug-resistant Tuberculosis (DR-TB) patients. TB management issues ranging from side effects of TB medication, disruption of treatment and lack of psychosocial support may increase the severity of depression in persons with drug-resistant TB.

“*The patients majorly have depression and anxiety*. *These issues manifest mostly after diagnosis once they are confirmed to have TB and are told of the treatment process*.*”- County Director of Health*

“*The patients majorly have Depression and stress caused by the duration within which they need to take the drugs*.*”- County TB coordinator*.

“*We have also seen patients who are DR-TB who have gone into depression because of abandonment by their partners*. *They are seen as outcasts*. *Depression is common among female patients”-Sub County TB Coordinator*

#### Subtheme 2: Coping mechanisms

A common coping mechanism for TB patients as observed by Key informant Interview respondents was drug abuse.

“*Alcoholism and Drug abuse come in as a coping mechanism for depression and or anxiety*.*”- County Director of Health*

“*In Ndhiwa*, *drug abuse is rampant*. *They resort to drugs to make them forget what they are going through”-Sub County TB Coordinator*

#### Subtheme 3: Psychological trauma

FGD respondents stated that most patients undergo anxiety and or depression during the presumptive stage of TB. They can’t tell what they are suffering from; this can be very stressful. Further, psychosis was found to present mostly during the treatment phase because of anti-TB medications.

“*When patients witnessed the symptoms*, *they assumed they were infected with HIV and AIDS*. *Additionally*, *people who don’t understand think the patients are taking ARVs*, *which affects the patients psychologically*.*”- Relapsed TB patient*, *Kakamega*

“*The patients also get psychological trauma when the diagnosis is confirmed*, *where the patients experience denial and seek second opinions*. *We have a few patients because of the drugs used to manage*, *especially MDR; there have been two psychotics however*, *normalcy was restored when the drugs were withdrawn*.*”- County TB coordinator*.

### Theme 3: Community perception, support and education

#### Subtheme 1: Community misinformation

FGD respondents stated that they received support from family and friends throughout their treatment. This involved constant encouragement, reminders to take their drugs in time, and financial aid when there is a loss of employment. However, in some instances, the patients were stigmatized by the community members and family due to the misinformation concerning TB. This stigma impacts their mental health and leads to poor outcomes.

“*Uneducated community members who mostly misinterpret and speculate negatively about TB spread stigma among the patients*, *this can lead to suicidal tendencies*, *depression*, *poor nutrition and high TB mortality rates in such communities”-* ***Current patient*, *Homabay***

Healthcare workers suggested that cultural beliefs as well as an association of TB with HIV are the key factors that can cause marginalization and social exclusion of TB Patients. It is regarded that HIV-related stigma automatically extends to TB patients.

“*TB is associated with witchcraft; this affects them in terms of seeking treatment services and adherence to treatment*. *It also affects their self-worth and interaction with others in the society”-* ***Sub-county MOH Official***

#### Subtheme 2: Community education and advocacy

The KII and FGD respondents stated that sensitization of community members on mental health issues is critical to improving community support and reducing the stigmatization of TB patients.

“*When World TB Day is around*, *there is a lot of messaging on the prevention and treatment of TB; proper ventilation*, *cough hygiene”-****Relapsed Patient*, *Busia***

Also, the provision of information, Education, and Communication (IEC) materials on TB including signs and symptoms, will enable community members to seek healthcare services early. Further, a revival of support groups for TB patients is critical in all health facilities with a TB clinic.

“*Invest in advocacy*, *communication*, *and social mobilization so that people can be able to understand the key messages on TB*.*”-* ***County TB Coordinator*.**

### Theme 4: Healthcare worker attitudes and systemic challenges

#### Subtheme 1: Healthcare worker attitudes

Healthcare worker attitudes can significantly impact the quality of service offered which can have a resultant effect on patients’ mental health status, utilization of services and adherence to treatment.

“*Not all HCWs have a positive attitude towards TB patients*. *HCWs feel that they will contract TB from the patients*. *Many staff do not want to be associated with TB patients because of the fear of contracting TB*. *If the HCW has a negative attitude*, *then definitely they will not give quality service to the patient and as a result*, *this patient may not come for subsequent appointments/treatments*.*”-****Sub County TB coordinator***.

“*The HCWs receive the patients well and therefore*, *they influence the patients positively and encourage them to continue seeking treatment*. *The attitude affected the patient positively as the health worker did constant follow-ups and constant reminders on upcoming clinic visits*. *The HCWs also checked on the progress of the patient*.*”-* ***Current TB Patient -Kakamega***

#### Subtheme 2: Health worker capacity

The research sought to establish whether the healthcare workers were prepared and equipped to effectively manage TB patients with mental health issues. The majority of the KII respondents stated that HCWs were not prepared to handle TB patients’ mental health issues.

“*HCWs rely on the knowledge they got from medical school*, *but things are ever-changing in the medical field*. *For instance*, *one could be administering a drug for depression that is no longer effective*.*”-****Sub County TB coordinator***

HCWs felt that there was an urgent need to strengthen their capacity. They proposed the use of refresher courses, on-job training (OJTs) and Continuous Medical Education (CMEs) to enhance their skills and capacity to diagnose and manage mental health issues among TB patients.

“*All HCW workers have some medical background to handle mental health issues*. *We need further sensitization and refresher training*. *Generally*, *mental health issues have not been investigated even among the staff themselves*.*”-****Sub County TB coordinator*.**

#### Subtheme 3: Systemic challenges

Under-resourced health care was identified as a major obstacle in delivering quality care to TB patients, ranging from inadequate diagnostic capacity, lack of drugs, and lack of funds to conduct training.

“*The knowledge is there but the availability of drugs for treatment is the problem*.*”-****Sub County TB coordinator*.**

“*Engage more mental health practitioners so that they can be accessible to the patient as and when need be*.*”-* ***County TB Coordinator***

Improved TB preventive and control measures, especially those targeting the social determinants of health are significant steps in reducing the impact of TB and mental issues in our society. A surge in TB infection is always associated with inaccessible TB diagnostic services, an uneducated society, and poor uptake of TB preventive services (e.g. BCG vaccines).

## 4 Discussion

The study sought to establish the prevalence of mental health issues among TB patients.

Depression and anxiety were the most common mental health issues experienced by TB patients across all the stages of TB management. This is consistent with a study by Sweetland et al., [15] in which depression was estimated to be as high as 48.9% among individuals with tuberculosis. Elevated depression and anxiety scores were associated with an increase in the number of TB symptoms reported by TB patients [16]. A significant number of TB patients acknowledged using alcohol as a coping mechanism. Alcohol can impair a patient’s judgement, leading to missed clinic appointments and non-adherence to the treatment regimen. Past studies indicate that individuals with an alcohol use disorder have a significantly higher likelihood of developing active TB, experiencing TB re-infection, and treatment non-adherence [17, 18].

From the study findings, stigma was the main barrier to TB management. This is consistent with a study that found a link between stigma and TB treatment outcomes [19]. Negative societal beliefs about TB and HIV co-morbidities appear to contribute to TB stigmatization in society [20].

Because of such beliefs, community members don’t always accept TB patients. This, coupled with self-stigmatization, leads to reduced self-esteem among TB patients and explains why patients may not disclose their TB status due to shame or guilt, thus increasing the risk of transmission of TB in the community. This finding is comparable to another study in Ghana [21]. TB diagnosis is associated with a loss of self-esteem, fears of rejection and infecting loved ones.

The survey sought to establish the existing support structures for TB patients with mental health issues. Most patients could seek support from their family members or the healthcare system. This support motivated the patients and kept them committed to their healing journey. A similar study conducted among patients with multidrug-resistant tuberculosis (MDR-TB) in Nepal highlighted the importance of psychosocial support packages in ensuring adherence to the complicated MDR-TB treatment regimen [22]. Negative attitudes and lack of support from family, health service providers and the wider community are significant barriers to ensuring continuity of treatment [23].

The study also assessed the impact of HCWs’ attitudes and perception on the quality of care, treatment adherence and loss of follow-up, with the findings indicating that HCWs’ attitudes could impact the mental health of TB patients, causing them to refrain from seeking health services which can lead to relapsing and defaulting. Negative attitudes towards TB patients by some HCWs, as evidenced by studies in Nepal [24] and Ethiopia [25] can impact treatment outcomes. There was a perceived lack of knowledge and skills among HCWs. This can lead to signs of mental health issues being missed, which may result in substandard patient care. A study by Galea et al. [26] concluded that mental health care should be integrated into TB programs as such integration can enhance the quality of services and bridge the gap in mental health care.

## 5 Conclusion

In conclusion, the study highlights the high prevalence of mental health issues, particularly anxiety and depression, among TB patients at all stages of TB management. Stigma was identified as a significant barrier to TB management, with negative societal beliefs about TB and HIV comorbidities contributing to the stigmatization of TB patients. However, the study also found that patients with support from their families or the healthcare system were more committed to their treatment journey, highlighting the importance of psychosocial support structures in ensuring adherence to TB treatment. Negative attitudes towards TB patients from HCWs can further impact patients’ mental health and treatment outcomes. A lack of knowledge and skills among HCWs can result in missed signs of a mental health issue and substandard patient care.

Based on these findings, we recommend that healthcare systems prioritize integrating mental health care into TB programs to address the high prevalence of mental health issues among TB patients. This can include training HCWs to identify and manage mental health issues in TB patients, providing psychosocial support packages for TB patients, and raising awareness in the community to reduce stigmatization. By implementing these recommendations, we can improve the quality of care for TB patients, especially those with mental health issues, and reduce the burden of TB in the community.

## Supporting information

S1 File Final TB dataset with labels. (XLSX)

S2 File Data collection tools. (DOCX)

S1 Fig Patient TB diagnosis. (DOCX)

S2 Fig Impact of TB diagnosis on patients. (DOCX)

S3 Fig Effect of stigma on access and utilization of TB services. (DOCX)

S4 Fig Mental health issues that TB patients face. (DOCX)

S5 Fig Rate of MH issues at each stage of the TB management pathway. (DOCX)

S6 Fig Types of support offered by family and friends. (DOCX)

S7 Fig Available Support structures/systems for TB patients with MH issues. (DOCX)

S8 Fig How the community structures can be improved. (DOCX)

S9 Fig How the attitudes and perceptions of HCWs contribute to MH issues among TB patients. (DOCX)
