# Supplementary material for: Mental health issues associated with the management of tuberculosis in Homabay, Busia and Kakamega Counties, Kenya
Source: PLoS One. 2024 Apr 16;19(4):e0298268. doi: 10.1371/journal.pone.0298268 (PMC11020984; doi:10.1371/journal.pone.0298268)
Supplement: S2 File — (DOCX) [file pone.0298268.s002.docx]

Mental health issues associated with the management of tuberculosis in Homabay, Busia and Kakamega Counties, Kenya

TOOL 1: TB- Patient Survey Tool

Section 1: Demographics (Respond to all Questions)

| 1.1 | Consent notes of respondent | Agree 2. Not Agree |
| --- | --- | --- |
| 1.2 | Date of Interview |  |
| 1.3 | Age of Respondent (Actual) |  |
| 1.4 | Sex of respondent (Observe) | Male  Female |
| 1.5 | Respondent level of education | Never gone to school  Primary  Secondary  College  University  Postgraduate |
| 1.6 | Respondent marital status | Married  Single  Divorced/ separated  Widowed/ Widower |
| 1.7 | County | Bungoma  Busia  Homabay  Kakamega  Migori  Siaya |
| 1.8 | How long have you been on TB treatment | _____ months |
| 1.9 | Have you been diagnosed with TB before? | Yes/No |
| 1.10 | Are there any members of your household who have been diagnosed with TB? | Yes/No |
| 1.11 | Mode of transport to health facility | Walking  Cycling  Public transport  Private vehicle  Other (specify) |

Section II: Mental Health and TB

| 2.1 | What mental health (MH) issues did you face as a TB patient?  *(tick all that apply)* | Anxiety  Depression  Psychosis  Drug abuse  Trauma-related disorder  Alcoholism,  Tobacco-related  None  Don’t Know |
| --- | --- | --- |
| 2.2 | At what time of the TB management Pathway/Journey are MH issues High? | Suspicion  Testing  Management  Recovery  Don’t Know |

2.3 Which mental health issues did you face at each stage? *Tick all that apply*

| Mental Health issue | Suspicion | Testing | Management | Recovery | None | Don’t Know |
| --- | --- | --- | --- | --- | --- | --- |
| Anxiety |  |  |  |  |  |  |
| Depression |  |  |  |  |  |  |
| Psychosis |  |  |  |  |  |  |
| Drug abuse |  |  |  |  |  |  |
| Trauma-related disorder |  |  |  |  |  |  |
| Alcoholism |  |  |  |  |  |  |
| Tobacco-related |  |  |  |  |  |  |
| None |  |  |  |  |  |  |
| Don’t Know |  |  |  |  |  |  |

| 2.4 | What support did you receive from immediate family/friends to support MH issues?  *(Tick all that apply)* | Encouragement  Financial support  Reminder on taking drugs  Accompaniment to the clinic |
| --- | --- | --- |
| 2.5 | How does MH issues affect access and Utilization of TB services?  *(Tick all that apply)* | Non-adherence to anti-TB medication  Self-stigma  Isolation  Poor nutrition  Delay seeking treatment  Suicidal Thoughts and self-harm  Don’t Know |
| 2.6 | How does stigma impact on TB patients?  *(Tick all that apply)* | Delay in seeking treatment  Defaulting on treatment  Relapse  Limited access to services |
| 2.7 | What other issues affected your mental health during the course of TB management?  (*Tick all that apply)* | Stigma  Healthcare worker attitude  Pressure from life events  Employment status  Income  Marital status  Social relationships  Duration of treatment |

Section III: Health care structures/systems and mental health of TB patients’ management

| 3.1 | Are there any support structures/systems existing for TB patients with MH issues?  *(Tick all that apply)* | CHVs  Family  Support groups  Counselling centres  Treatment centres  Don’t Know |
| --- | --- | --- |
| 3.2 | How can the community structures be improved to enhance health seeking behaviors among TB patients with mental health issues?  *(Tick all that apply)* | Training CHVs and Health care workers on Mental health  Sensitizing community members on Mental Health  Integrating mental health into TB case management process  Establishment of counselling and treatment centres  Creation of a functional referral pathways |
| 3.3 | What TB Preventive measures are in place at the community level?  *(Tick all that apply)* | Sensitization on TB through Media and CHVs  TB education for the community  Accessibility of TB diagnosis services  CHVs referring suspected cases  Administering the BCG vaccine  Don’t know |
| 3.4 | How do the attitudes and perceptions of HCWs contribute to mental health issues among TB patients?  *(Tick all that apply)* | Stigmatization  Poor quality of care  Denial of services  Loss to follow up  Disengagement of the patient on TB care  Don’t Know |

Tool 2: Current TB patients FGD Guide

Personal level

1. What mental health (MH) issues do TB patients face? *Probe for depression, psychosis, anxiety, trauma-related disorder, alcoholism, tobacco-related, drug abuse etc.*
2. How does stigma impact on TB patients? *Probe for adherence, relapse, access to services etc.*
3. How do MH issues affect your access and use of TB services? *Probe for self esteem etc.*
4. Does the perception and attitudes of HCWs towards TB patients’ impact on their mental health and access to services?
5. What other issues affect your mental health during the course of TB management?

Community level

1. What are the existing community structures that support TB patients to seek mental healthcare services? Which structures don’t support?
2. How can the community structures be improved to enhance health seeking behaviors among TB patients with mental health issues?
3. What TB Preventive measures are in place at the community level?

Health facility level

1. How do the attitudes and perceptions of HCWs contribute to mental health issues among TB patients?

Tool 3: Recovered TB patients FGD Guide

Personal level

1. What mental health (MH) issues did you face as a TB patient? *Probe for depression, psychosis, anxiety, trauma-related disorder, alcoholism, tobacco-related, drug abuse etc.*
2. How does stigma impact on TB patients? *Probe for adherence, relapse, access to services etc.*
3. How did MH issues affect your access and use of TB services? *Probe for self-esteem etc.*
4. Does the perception and attitudes of HCWs towards TB patients’ impact on their mental health and access to services?
5. Was there any mental health support you received in the course of treatment that aided the recovery process?
6. What other issues affected your mental health during the course of TB management?

Community level

1. What existing community structures support TB patients to seek mental healthcare services? Which structures don’t support?
2. How can the community structures be improved to enhance health seeking behaviors among TB patients with mental health issues?
3. What TB Preventive measures are in place at the community level?

Health facility level

1. How do the attitudes and perceptions of HCWs contribute to mental health issues among TB patients?

Tool 4: Relapsed TB patients FGD Guide

Personal level

1. What mental health (MH) issues did you face as a TB patient? *Probe for depression, psychosis, anxiety, trauma-related disorder, alcoholism, tobacco-related, drug abuse etc.*
2. How does stigma impact on TB patients? *Probe for adherence, relapse, access to services etc.*
3. How did MH issues affect your access and use of TB services? *Probe for self-esteem etc.*
4. What factors other than mental health contributed to your TB management relapse?
5. If you went back to TB treatment, what factors contributed to your return? If not, what issues are preventing you from going back to TB treatment?
6. Does the perception and attitudes of HCWs towards TB patients’ impact on their mental health and access to services?
7. What other issues affected your mental health during the course of TB management?

Community level

1. What existing community structures support TB patients to seek mental healthcare services? Which structures don’t support?
2. How can the community structures be improved to enhance health seeking behaviors among TB patients with mental health issues?
3. What TB Preventive measures are in place at the community level?

Health facility level

1. How do the attitudes and perceptions of HCWs contribute to mental health issues among TB patients?

Tool 5: Health Care Workers KII Guide

Personal level

1. What mental health (MH) issues do TB patients face? *Probe for depression, psychosis, anxiety, trauma-related disorder, alcoholism, tobacco-related, drug abuse etc.*
2. How does stigma impact on TB patients? *Probe for adherence, relapse, access to services etc.*
3. How do MH issues affect your access and use of TB services? *Probe for self-esteem etc.*
4. Does the perception and Attitudes of HCWs towards TB patients’ impact on their mental health and access to services?

Community level

1. What are the existing community structures that support TB patients to seek mental healthcare services? Which structures don’t support?
2. What strategies can the county/sub-county employ to improve community structures to enhance mental health management of TB patients?
3. What TB Preventive measures are in place at the community level?

Health facility level

1. How have health care workers (HCWs) been prepared to effectively manage mental health issues in TB patients? What elements of mental health in TB management do HCWs need more support? *Probe for training and supportive supervision.*
2. How does HCW attitude and perception contribute to mental health issues among TB patients?
3. What strategies can be deployed to empower HCWs to provide optimal mental health support for TB patients? (*integrating TB and mental health etc.)*

National/County/Sub-county level (Policy)

1. Are TB treatment guidelines available at the health facilities and what are the levels of compliance in their use?
2. Are the current TB data management systems adequate enough to collect, collate and report on TB mental health issues?
3. What improvements can/should be made to the current TB data management systems adequate enough to collect, collate and report on TB mental health issues?
