# Supplementary figures and images for: Mental health issues associated with the management of tuberculosis in Homabay, Busia and Kakamega Counties, Kenya
Source: PLoS One. 2024 Apr 16;19(4):e0298268. doi: 10.1371/journal.pone.0298268 (PMC11020984; doi:10.1371/journal.pone.0298268)

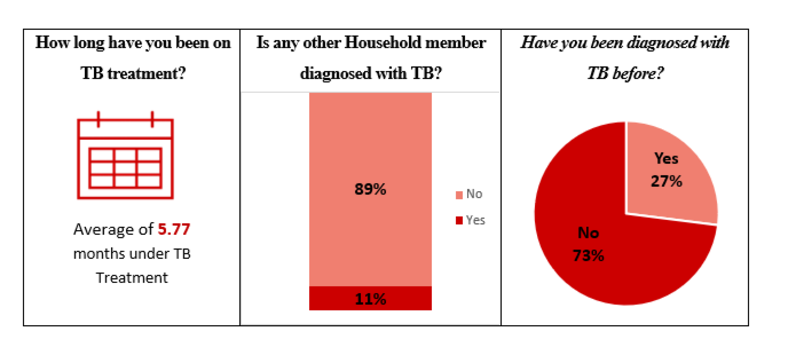


**S1 Fig: Patient TB diagnosis**

Supplement: S1 Fig — (DOCX) [file pone.0298268.s003.docx]

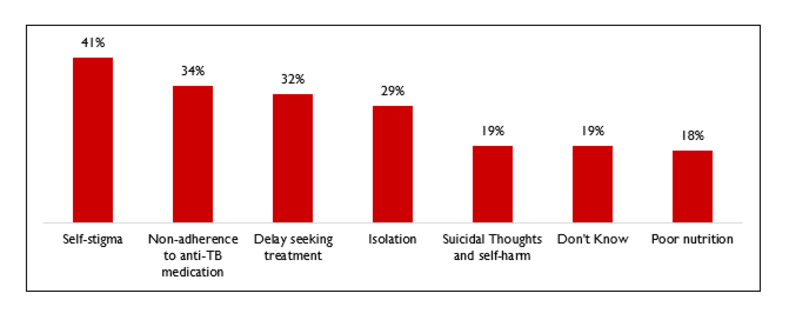


**S2 Fig: Impact of TB diagnosis on patients**

Supplement: S2 Fig — (DOCX) [file pone.0298268.s004.docx]

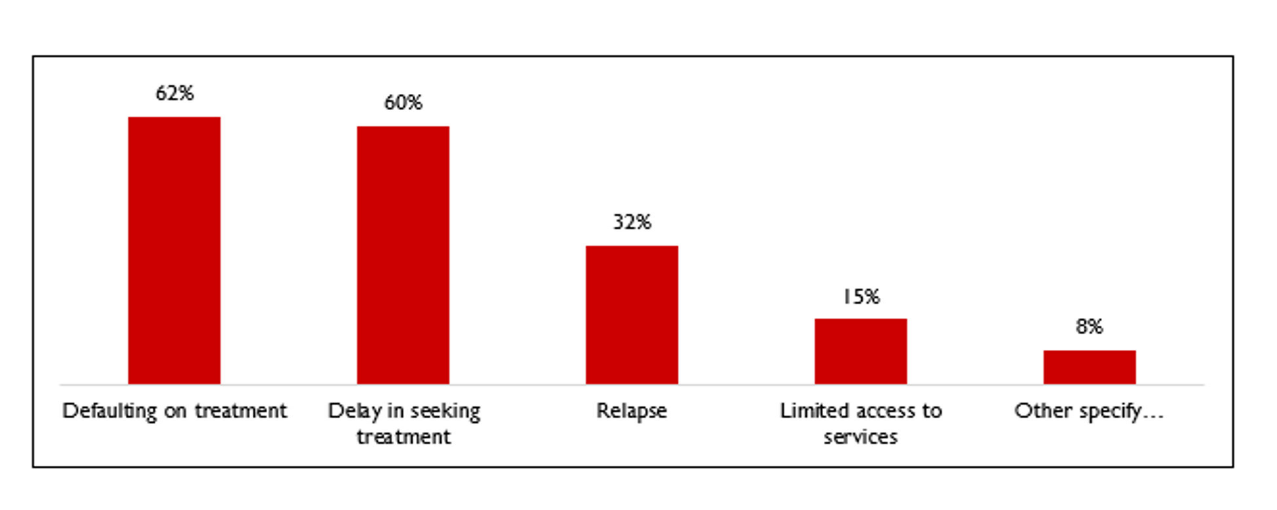


**S3 Fig: Effect of stigma on access and utilization of TB services**

Supplement: S3 Fig — (DOCX) [file pone.0298268.s005.docx]

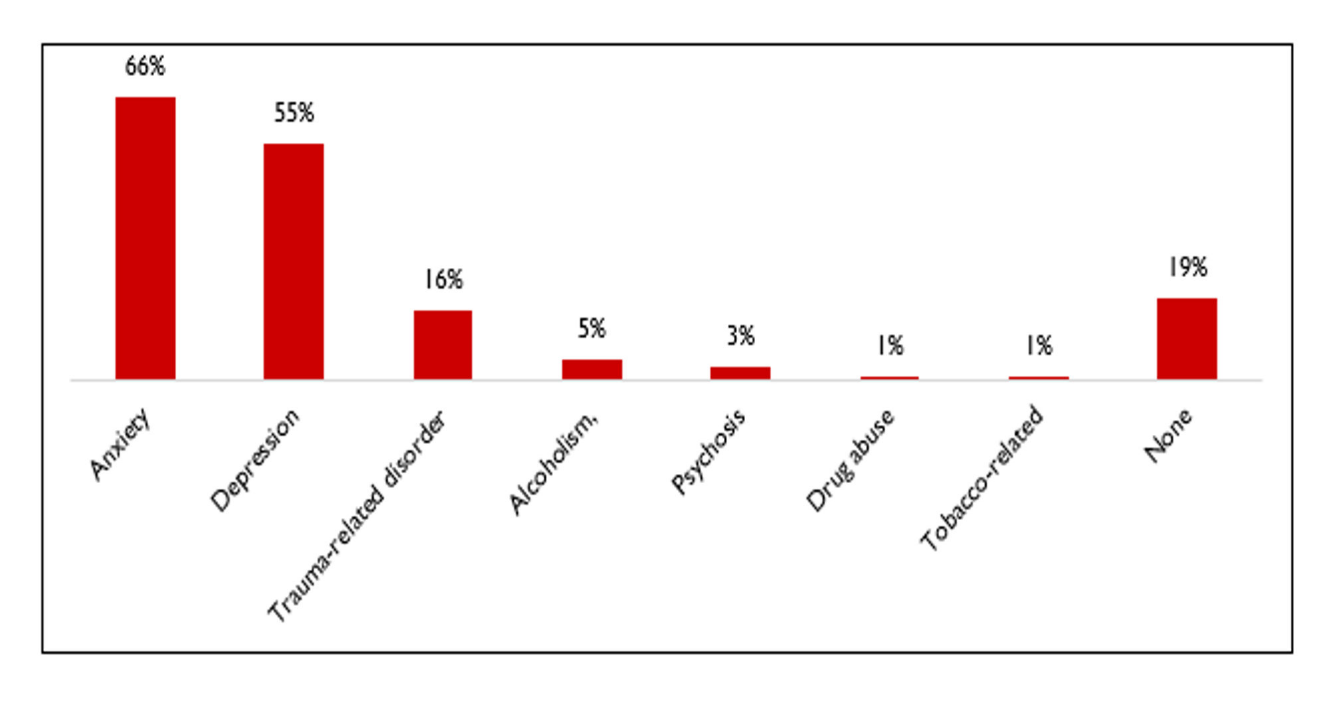


**S4 Fig: Mental health issues that TB patients face**

Supplement: S4 Fig — (DOCX) [file pone.0298268.s006.docx]

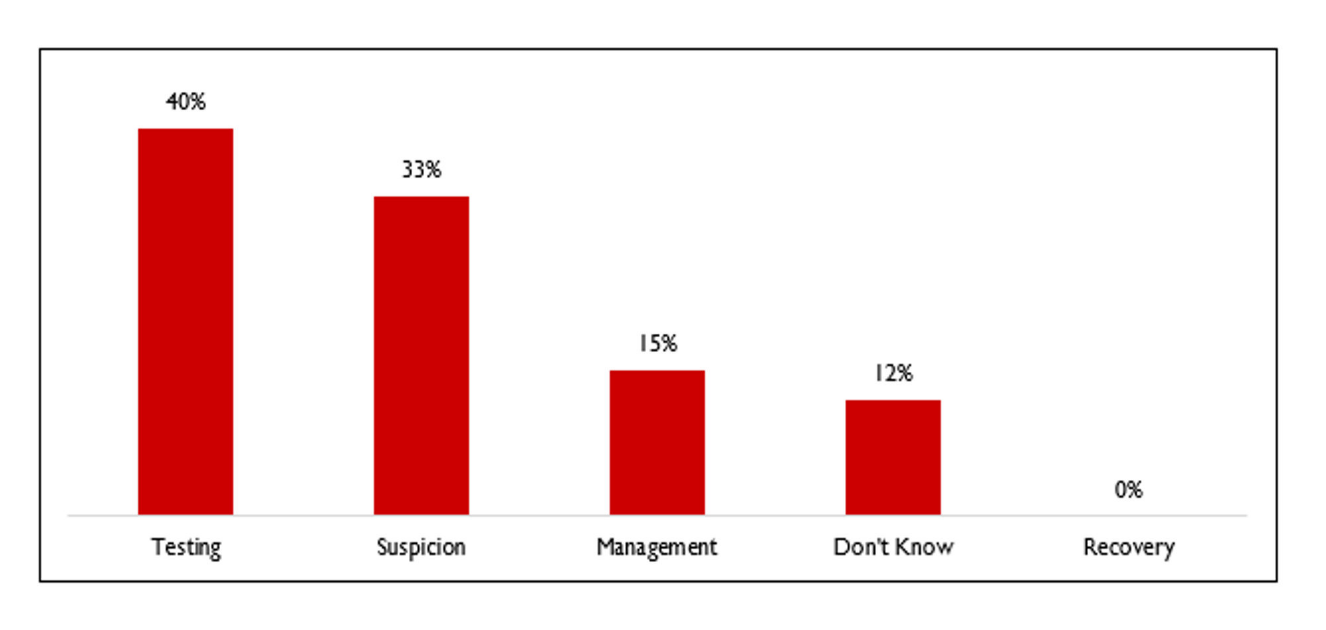


**S5 Fig: Rate of MH issues at each stage of the TB management pathway**

Supplement: S5 Fig — (DOCX) [file pone.0298268.s007.docx]

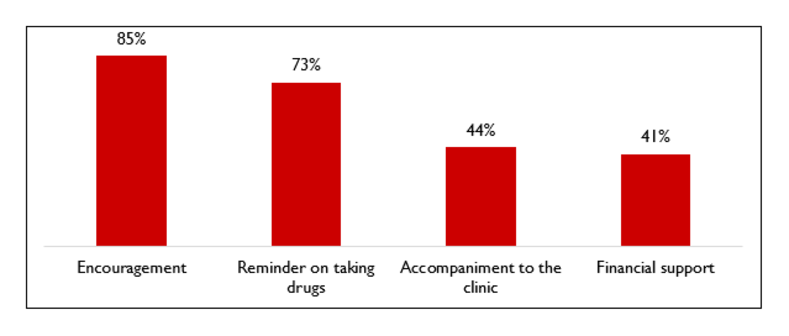


**S6 Fig: Types of support offered by family and friends**

Supplement: S6 Fig — (DOCX) [file pone.0298268.s008.docx]

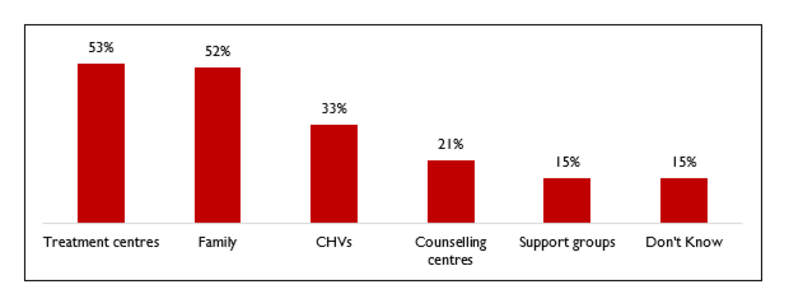


**S7 Fig: Available Support structures for TB patients with MH issues**

Supplement: S7 Fig — (DOCX) [file pone.0298268.s009.docx]

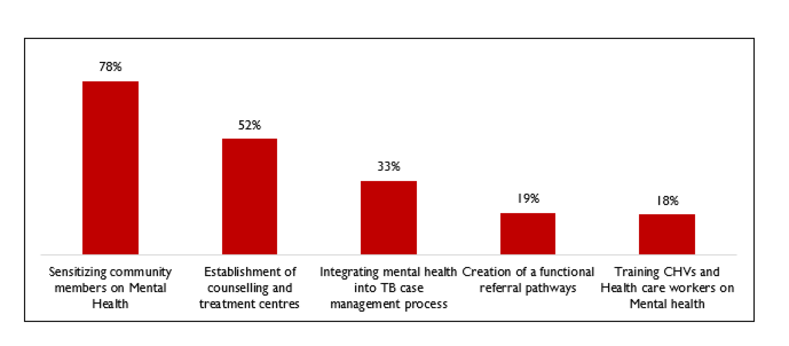


**S8 Fig: How the community structures can be improved**

Supplement: S8 Fig — (DOCX) [file pone.0298268.s010.docx]

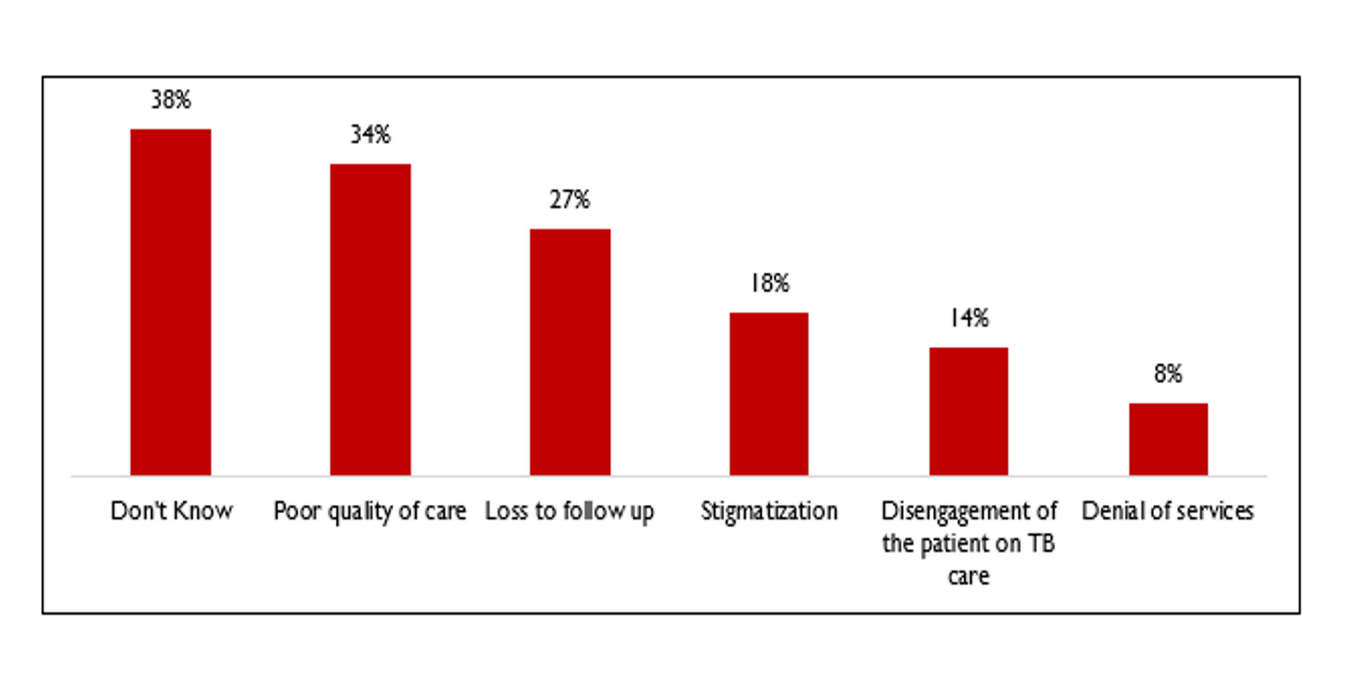


**S9 Fig: How the attitudes and perceptions of HCWs contribute to MH issues among TB patients**

Supplement: S9 Fig — (DOCX) [file pone.0298268.s011.docx]
